# Circulatory zinc transport is controlled by distinct interdomain sites on mammalian albumins†
†Electronic supplementary information (ESI) available. See DOI: 10.1039/c6sc02267g



**DOI:** 10.1039/c6sc02267g

**Published:** 2016-08-15

**Authors:** Katarzyna B. Handing, Ivan G. Shabalin, Omar Kassaar, Siavash Khazaipoul, Claudia A. Blindauer, Alan J. Stewart, Maksymilian Chruszcz, Wladek Minor

**Affiliations:** a Department of Molecular Physiology and Biological Physics , University of Virginia School of Medicine , PO Box 800736 , Charlottesville , VA 22908-0736 , USA . Email: wladek@iwonka.med.virginia.edu ; Tel: +1-434-243-6865; b New York Structural Genomics Research Consortium (NYSGRC) , USA; c School of Medicine , University of St. Andrews , St. Andrews KY16 9TF , UK; d Department of Chemistry , University of Warwick , Coventry CV4 7AL , UK; e Department of Chemistry and Biochemistry , University of South Carolina , Columbia , South Carolina 29208 , USA

## Abstract

Zinc is an essential nutrient in the body; it is required for the catalytic activity of many hundreds of human enzymes and virtually all biological processes, therefore its homeostasis and trafficking is of crucial interest. Serum albumin is the major carrier of Zn^2+^ in the blood and is required for its systemic distribution. Here we present the first crystal structures of human serum albumin (HSA) and equine serum albumin (ESA) in complex with Zn^2+^. The structures allow unambiguous identification of the major zinc binding site on these two albumins, as well as several further, weaker zinc binding sites. The major site in both HSA and ESA has tetrahedral geometry and comprises three protein ligands from the sidechains of His67, His247 and Asp249 and a water molecule. Isothermal titration calorimetric studies of a HSA H67A mutant confirm this to be the highest affinity Zn^2+^ site. Furthermore, analysis of Zn^2+^ binding to HSA and ESA proved the presence of secondary sites with 20–50-fold weaker affinities, which may become of importance under particular physiological conditions. Both calorimetry and crystallography suggest that ESA possesses an additional site compared to HSA, involving Glu153, His157 and His288. The His157 residue is replaced by Phe in HSA, incapable of metal coordination. Collectively, these findings are critical to our understanding of the role serum albumin plays in circulatory Zn^2+^ handling and cellular delivery.

## Abstract 2

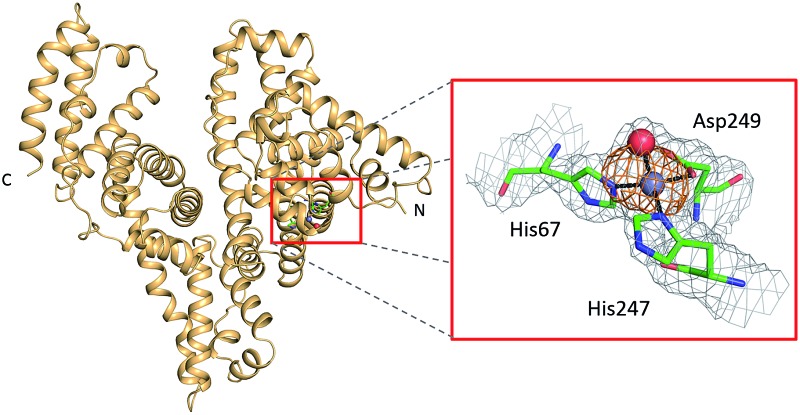
Circulatory transport of the essential nutrient zinc primarily occurs through its binding to serum albumin. Here, we present the first crystal structures of mammalian albumins in complex with zinc. These structures, together with accompanying zinc binding data, allow identification of key zinc transport sites on human and equine albumins.

## Introduction

Serum albumin is an abundant plasma transport protein (*ca.* 600 μM), which is composed of three homologous domains. It is known to bind and transport a wide variety of molecules including fatty acids, hormones, drugs and metal ions.^1,2^ The ability to bind such a vast and diverse group of molecules is reflected in its flexible three-dimensional structure.^3^ Even in the absence of ligands, albumin is constantly sampling available conformational space in a process which has been described as “breathing”.^1^ Among many physiologically important molecules, albumin is the major carrier of Zn^2+^ in blood.^4^ Zn^2+^ is crucial for the activity of many hundreds of extra- and intracellular enzymes and transcription factors. Approximately 75–80% of plasma Zn^2+^ (∼14 μM) is bound to albumin, accounting for as much as 98% of the exchangeable fraction of Zn^2+^ in blood plasma.^5^ Serum albumin effectively acts as an extracellular “zinc buffer” that controls the concentrations of “free” Zn^2+^ ions that are available to other plasma proteins or for cellular uptake through membrane-bound zinc transporters.^6^ Thus, serum albumin not only controls the uptake of Zn^2+^ into cells including endothelial cells^7^ and erythrocytes,^8^ but also protects blood cells and the endothelial cells which line the blood vessels from the otherwise toxic concentration of Zn^2+^ in plasma.^9,10^ Additionally, receptor-mediated transport of Zn^2+^ across the endothelium has been demonstrated with albumin–Zn^2+^ complexes *in vitro*.^11^

Serum albumin is also involved in the handling of Cu^2+^, Ca^2+^ and Mg^2+^ in mammals, helping to control the biologically active levels of these metals in the blood.^12^ Significantly, recent work has demonstrated that there is cross-talk between the binding of Zn^2+^ and free fatty acids, turning albumin into a device that links plasma Zn^2+^ speciation with energy metabolism.^13,14^ Knowledge of the structural features that mediate this cross-talk is essential for understanding and influencing this metabolic link.

Based on equilibrium dialysis experiments and ^113^Cd NMR spectroscopic studies, two or three high-affinity Zn^2+^-binding sites have been suggested to exist on bovine (BSA) and human (HSA) serum albumins.^15–18^ One site is known to have considerably higher affinity towards Zn^2+^ than any other site(s); this site is known as site A or the multi-metal binding site.^19^ For HSA, the location of this site at a domain interface was suggested through site-directed mutagenesis and ^111^Cd NMR spectroscopy,^20^ and subsequent EXAFS and molecular modeling studies indicated the participation of His67 and Asn99 from domain I, and His247 and Asp249 from domain II.^9^ Besides Zn^2+^, this site can also bind other metal ions including Cu^2+^, Ni^2+^, Cd^2+^,^19^ and Co^2+^.^21^ A second putative Cd^2+^/Zn^2+^-binding site on various albumins is called site B;^17^ the existence of this site has been demonstrated by ^111/113^Cd NMR spectroscopy,^15,17,20,22^ but its location has remained unknown. A third, weak site for both Cd^2+^ and Zn^2+^ has been suggested by equilibrium dialysis,^22^ but this has not been observed by any other technique. In addition, up to a further nine still weaker sites were detected by gel filtration chromatography.^23^ At present there is no evidence relating to the locations of any Zn^2+^ sites on albumin other than site A. These secondary Zn^2+^ sites on serum albumin extend the zinc binding capacity of albumin and may be biologically important, especially when site A is impacted by fatty acid binding^13,14^ and/or the Zn^2+^ concentration is very high.

Despite the critical role of albumin in the circulatory handling of Zn^2+^, there are no structural data related to specific binding sites on serum albumin and their geometry in the Protein Databank (PDB). Furthermore, only in 2012 were structures of serum albumins from mammalian species other than humans deposited in the PDB.^24,25^ Here, we present the first crystal structures of human serum albumin (HSA) and horse serum albumin (ESA) in complex with Zn^2+^ at different concentrations. We discuss the location, geometry, and residues involved in metal coordination in several Zn^2+^-binding sites. The crystallographic studies, together with results from isothermal titration calorimetry (ITC), provide conclusive evidence for the location of the major zinc site in both human and horse albumins. Furthermore, both structural and ITC analysis revealed differences in Zn^2+^ binding at secondary sites between the two albumins, suggesting that circulatory Zn^2+^ handling may differ between the two species.

## Results

### Overall albumin structure

**Fig. 1:**
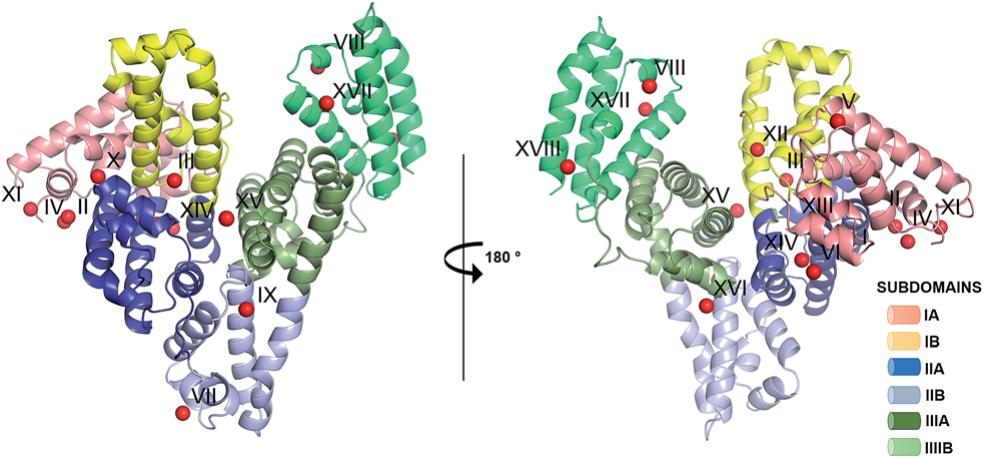
Crystal structure of ESA in complex with Zn^2+^. All structures of ESA–Zn^2+^ presented herein are superposed and a single Zn^2+^ for each site is shown as a representative. The overall fold in the ; 5IIH structure is shown as representative. Helices are represented by ribbons and Zn^2+^ by red spheres. Zn^2+^ binding sites are marked with numbers I–XVIII as in Table 3.

One HSA and five ESA crystal structures in complex with Zn^2+^, obtained by co-crystallization and soaking, are presented here (Table 1 and 2). In general terms, HSA and ESA structures are highly similar, as may be expected given the 76.2% sequence identity and 84.8% sequence similarity between HSA and ESA. All six structures resemble the canonical “heart shape” composed of helices and loops (Fig. 1). Each structure can be divided into three homologous domains (from now on residue numbering will refer to HSA): I (residues 1–195), II (196–383) and III (384–585), each containing two sub-domains (A and B) composed of four and six alpha-helices, respectively (Fig. 1). Structures are additionally stabilized by 17 conserved disulfide bridges. The refined crystallographic models display good overall geometry with a low percentage of rotamer outliers and a low MolProbity clash score (Table 2).

**Table 1:** Crystallization and cryo-protection conditions for the crystal structures of HSA and ESA in complex with Zn^2+^

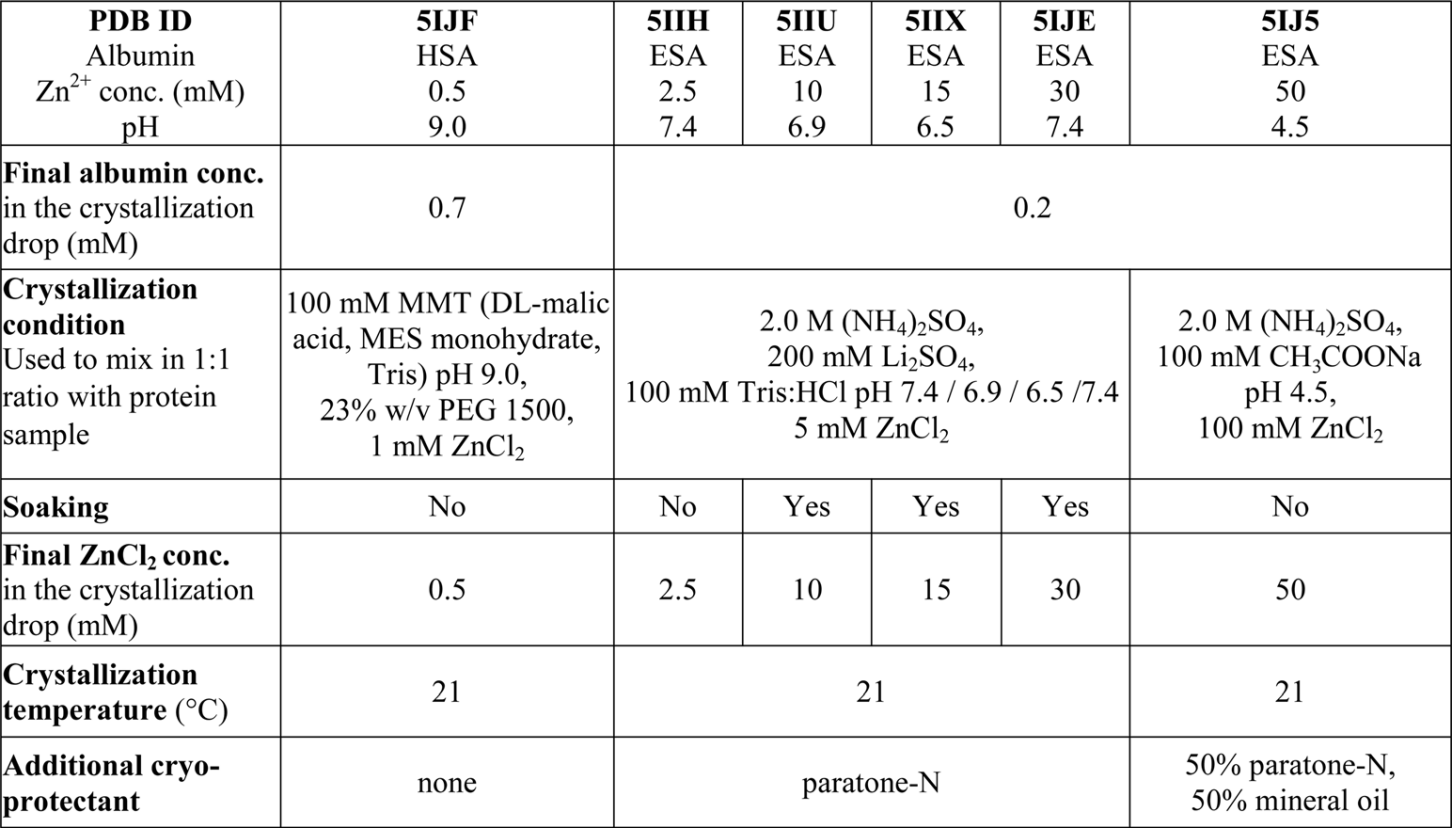

**Table 2:** Data collection and refinement statistic for HSA–Zn^2+^ and ESA–Zn^2+^ structures. Values in parentheses are for the highest resolution shell

**PDB ID**	** 5IJF **	** 5IIH **	** 5IIU **	** 5IIX **	** 5IJE **	** 5IJ5 **
Albumin	HSA	ESA	ESA	ESA	ESA	ESA
Zn^2+^ conc. (mM)	0.5	2.5	10	15	30	50
pH	9.0	7.4	6.9	6.5	7.4	4.5

**Data collection**						
Wavelength (Å, keV)	0.979, 12 664	0.979, 12 664	0.979, 12 664	0.979, 12 664	1.282, 9668, 1.289, 9618	0.979, 12 664
Space group	*C*222_1_	*P*6_1_	*P*6_1_	*P*6_1_	*P*6_1_	*P*6_1_
*Unit-cell parameters*						
*a*, *b*, *c* (Å)	78.6, 121.6, 140.0	93.10, 93.10, 141.44	93.75, 93.75, 141.79	94.31, 94.31, 141.49	94.38, 94.38, 141.91	96.13, 96.13, 144.15
*α*, *β*, *γ* (°)	90.0, 90.0, 90.0	90.0, 90.0, 120.0	90.0, 90.0, 120.0	90.0, 90.0, 120.0	90.0, 90.0, 120.0	90.0, 90.0, 120.0
Resolution range (Å)	50.0–2.65 (2.70–2.65)	50.0–2.40 (2.44–2.40)	80.0–2.30 (2.34–2.30)	80.0–2.20 (2.24–2.20)	50.00–2.40 (2.40–2.44)	50.00–2.55 (2.55–2.59)
Completeness (%)	98.9 (88.4)	99.6 (99.3)	99.3 (99.0)	100 (100)	99.5 (99.9)	99.9 (100.0)
Total number of reflections	143 674	208 207	300 971	277 833	120 428	151 772
No. of unique reflections	19 741	25 677	31 099	36 226	27 885	23 268
Mean *I*/*σ*(*I*)	28.7 (2.1)	32.2 (2.1)	29.1 (1.9)	31.1 (2.2)	23.0 (2.1)	23.8 (2.0)
CC1/2 – highest resolution shell	0.84	0.85	0.89	0.89	0.79	0.70
Redundancy	7.3 (6.6)	7.7 (7.7)	9.7 (9.4)	7.7 (7.5)	4.3 (4.1)	6.2 (5.7)
*R*_merge_ ^*a*^ (%)	7.1 (82.0)	7.1 (96.5)	7.8	8.9 (79.5)	7.9 (70.7)	0.107

**Structure refinement**						
*R*_work_ ^*b*^ (%)	21.3	17.5	17.9	17.8	19.5	18.9
*R*_free_ ^*b*^ (%)	29.2	23.5	23.9	22.8	25.5	25.3
Bond lengths RMSD (Å)	0.008	0.009	0.008	0.008	0.008	0.044
Bond angles RMSD (°)	1.2	1.2	1.2	1.2	1.2	1.2
Mean *B* value (Å^2^)	105	72	62	58	55	76
Number of protein atoms	4279	4501	4514	4484	4463	4411
Mean *B* value for protein atoms (Å^2^)	105	72	62	58	56	76
Number of water molecules	40	133	193	364	296	159
Mean *B* value for water molecules (Å^2^)	85	61	56	57	50	66
Number of ligands/ions atoms	9	6	15	50	18	21
Mean *B* value for ligands/ions atoms (Å^2^)	92	110	86	74	73	94
Clash score	0.97	0.79	0.79	0.45	0.92	0.58
Clash score percentile	100	100	100	100	100	100
Rotamer outliers (%)	0.24	0.83	0.83	0.21	0.64	0.66
Ramachandran outliers (%)	0.00	0.00	0.00	0.00	0.00	0.00
Ramachandran favored (%)	97.71	98.27	97.93	98.25	98.62	98.10
Residues with bad bonds (%)	0.00	0.00	0.00	0.00	0.00	0.00
Residues with bad angles (%)	0.00	0.00	0.00	0.00	0.00	0.00
MolProbity score	0.85	0.75	0.77	0.66	0.78	0.69

^*a*^ 
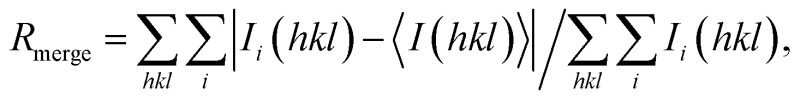
 where *I*(*hkl*) is the mean of *I* observations *I*_*i*_(*hkl*) of reflection *hkl*.

^*b*^ 
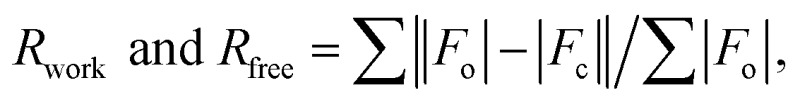
 where *F*_o_ and *F*_c_ are the observed and calculated structure factors, respectively, calculated for working set (*R*_work_) and for 5% of the data omitted from refinement (*R*_free_).

### Zinc detection and identification in the structures

The position and identity of the metal in each distinct metal binding site were confirmed by the characteristic anomalous scattering of zinc for both HSA and ESA structures with various approaches. First, for five of the structures (Table 2) the diffraction data were collected on the selenium absorption K-edge (12 664 eV), which is above the zinc absorption K-edge (9659 eV), ensuring significant signal from Zn^2+^ on anomalous electron density maps. The presence of anomalous peaks indicated the positions of Zn^2+^ in the unit cell and enabled correct placement of Zn^2+^ ions even in low resolution structures. Second, to confirm that the anomalous signal was produced by zinc, the X-ray fluorescence spectra at excitation wavelengths above (9668 eV) and below (9618 eV) the zinc absorption K-edge were recorded for the crystal with the highest Zn^2+^ concentration at neutral pH (Table 2, ; 5IJE). In the spectra at the excitation energy of 9668 eV there were two peaks, at 9600 and 8660 eV (Fig. S1†). The first peak corresponds to the energy of the incident X-ray beam. The peak at 8660 eV refers to the K-level emission line for zinc. Disappearance of the K-level zinc emission peak in the fluorescence spectrum collected at the excitation energy of 9618 eV proves that the anomalous scatterer is zinc. Third, for the same crystal (Table 2, ; 5IJE), two diffraction data sets were collected at energies above (9668 eV) and below (9618 eV) the zinc absorption K-edge. The subsequent calculation of the anomalous map showed that the strong anomalous peaks interpreted as Zn^2+^ in the first experiment (data collected above zinc absorption K-edge) disappeared in the second experiment, proving that the anomalous peaks were caused by the bound Zn^2+^. Given the similarity of experimental setup (same protein source and buffer solutions), we present here X-ray fluorescence scan results and diffraction data collected below the zinc absorption K-edge only for one crystal. However, at least 10 similar experiments for crystals originating from different crystallization plates and drops were performed to ensure the identity of the metal in the structures. Furthermore, no significant anomalous signal was detected in diffraction data collected at energies above the zinc absorption K-edge for ESA crystals produced without addition of ZnCl_2_, suggesting that all the significant anomalous signal observed in our structures is a result of addition of ZnCl_2_.

### Structure of HSA in complex with zinc

The crystal structure of defatted HSA in complex with Zn^2+^ was obtained by co-crystallization in the presence of 0.5 mM ZnCl_2_, and is the first crystal structure of HSA determined at basic pH (pH 9, Table 1). The crystal belonged to space group *C*222_1_ with one protein molecule in the asymmetric unit (Table 2). The same space group has been reported before only for the structure of HSA in complex with the albumin-binding module from *Finegoldia magna*, but with significantly different cell dimensions (1TF0).^26^ The structure was refined at 2.65 Å resolution. The HSA–Zn^2+^ complex adopts a conformation closely similar to the structure of native HSA (; 1AO6)^27^ crystallized in the *P*1 space group (C-alpha RMSD 0.7 Å, 571 atoms) as well as to the previously mentioned structure 1TF0 (C-alpha RMSD 1.0 Å, 556 atoms). The structure has a high mean atomic displacement parameter (B-factor) for the protein atoms – 90 Å^2^. The high B-factor can be explained by the well-known high structural flexibility of HSA, which results in a number of surface residues having uninterpretable or absent electron density for their sidechains. As previously observed, the N-terminal part of the protein is disordered^28^ and the two first amino acids, Asp1 and Ala2, cannot be unambiguously built.

The structure of HSA in complex with Zn^2+^ was crystallized at approximately 1 : 1 protein : zinc ratio (0.7 mM HSA and 0.5 mM ZnCl_2_, Table 1), and only one strong Zn^2+^-binding site was confidently detected (Table 3, site I). There was one additional anomalous peak between His9 and Asp13, at the interface of a crystal contact, but Zn^2+^ could not be confidently built in this position due to its location on the unit cell edge.

**Table 3:** Summary of metal binding sites that have at least one protein ligand in different structures of HSA and ESA in complex with Zn^2+^. ‘x’ indicates that site is present in the structure. ‘*’ indicates that anomalous signal is present in the site, but at least one of the coordinating residues observed in other structures is not present in Zn^2+^ vicinity due to disorder or different conformation of the sidechain. ‘#’ indicates that Zn^2+^ could not be modeled in the location, see text for details. Residue numbers refer to HSA; in case of non-conserved residues, the HSA amino acid is given in parentheses

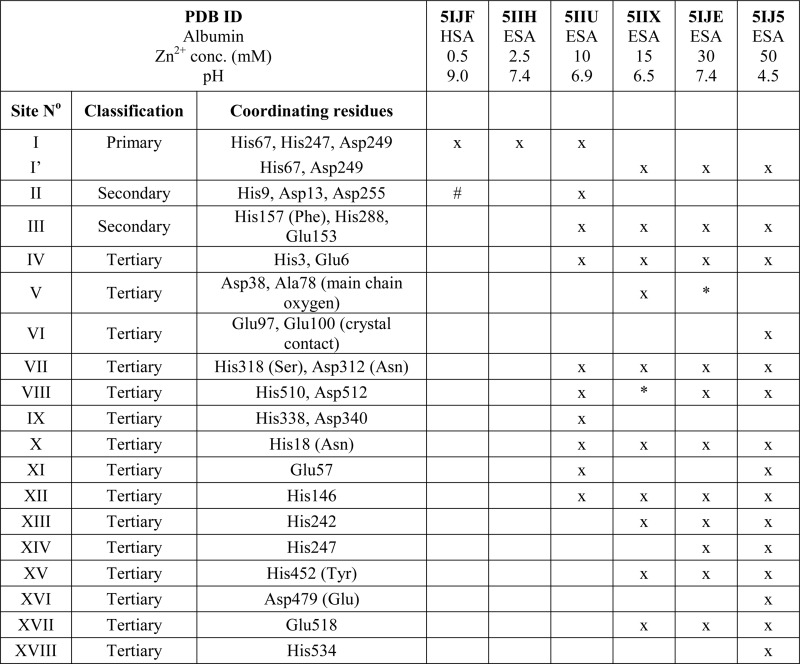

### Structures of ESA in complex with zinc

Five different complexes of defatted ESA with Zn^2+^ were determined in order to investigate the influence of pH and Zn^2+^ concentration on Zn^2+^-binding sites. The resolution of the structures spanned the range of 2.20–2.55 Å (Table 2). All structures of ESA–Zn^2+^ complexes were obtained by initial co-crystallization in the presence of 2.5 mM Zn^2+^ for the structures at near-neutral pH, and 50 mM Zn^2+^ for the structure at pH 4.5 (Table 1). Co-crystallization at neutral pH and higher zinc concentration did not produce diffraction quality crystals. In order to obtain crystals with higher zinc concentration, crystals obtained by initial co-crystallization in the presence of 2.5 mM Zn^2+^ were additionally soaked with ZnCl_2_ at final Zn^2+^ concentrations of 10, 15, and 30 mM resulting in structures ; 5IIU, ; 5IIX and ; 5IJE, respectively.

All five structures have the same space group *P*6_1_ and very similar unit cell parameters (Table 2) as the ESA apo form (; 3V08).^25^ The structures have one ESA molecule in the asymmetric unit and show similar protein conformation among each other as measured by low pairwise C-alpha RMSD values in the range of 0.31–0.66 Å. The C-alpha atoms superpositions of Zn^2+^-bound ESA with its apo form (; 3V08)^25^ shows RMSD values in the range of 1.10–1.16 Å, however when comparing to the structure at basic pH 9.0 (; 5HOZ), the RMSD value is much lower and varies between 0.31 and 0.67 Å. The average B-factor for protein atoms in Zn^2+^-bound structures varies in the range of 58–64 Å^2^. It was not possible to unambiguously build either two or three N-terminal residues in any of the structures because of the dynamic character of this protein region. Thus, the N-terminus of ESA seems to behave in a similar way to that of HSA.

In the structures of ESA in complex with Zn^2+^, the number of Zn^2+^ ions varied from 1 to 15 (Table 3). The varying number of occupied Zn^2+^-binding sites in the structures is related to changes in Zn^2+^ concentration (10 to 200-fold excess of Zn^2+^) and pH in crystallization conditions (Table 1). Zn^2+^ was placed only in sites that had a corresponding anomalous signal at least at the 4.0–5.3 σ level (depending on the structure). For most sites, we were able to determine complete Zn^2+^coordination spheres composed of atoms originating from protein sidechains and water molecules. In these cases, tetrahedral coordination of metal was observed. For 9 sites, strong anomalous peaks ensured Zn^2+^ placement, but the electron density map did not allow for confident building of full coordination spheres in at least one of the structures. There were additional peaks in all ESA structures on the verge of detection and with no detectable inner-sphere coordination, where Zn^2+^ ions could not be confidently placed.

### Zinc binding site A: geometry and dynamics

**Fig. 2:**
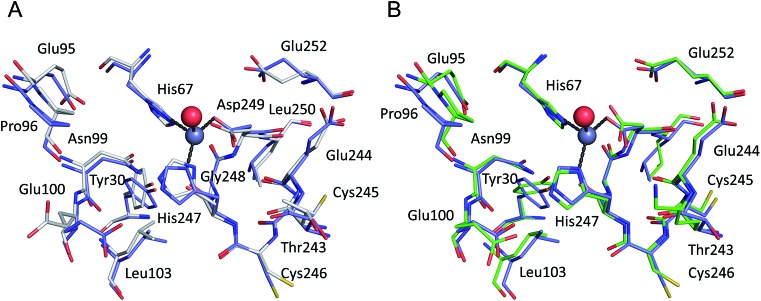
The all-atoms superposition of residues within 8 Å from Zn^2+^ in site a of HSA–Zn^2+^ complex (; 5IJF, blue), and corresponding residues in (a) native HSA structure (; 1AO6, white) and (b) ESA–Zn^2+^ structure (2.5 mM Zn^2+^, pH 7.4 – ; 5IIH, green). Zinc ions are shown in grey, oxygen in red, sulfur in yellow, nitrogen in dark blue, coordination bonds with Zn^2+^ ion are marked with black dashed lines. Residue numbers refer to HSA.

**Fig. 3:**
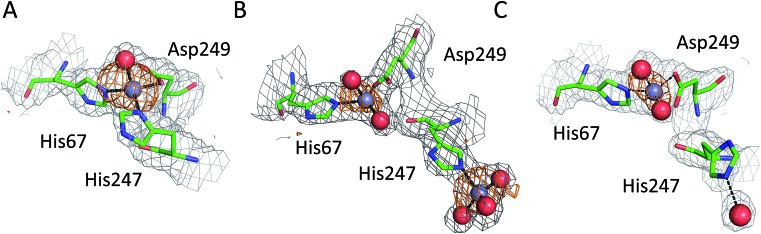
Representative structures showing dynamic behavior of His247 in ESA–Zn^2+^ complexes. (a) ESA–Zn^2+^, 2.5 mM Zn^2+^, pH 7.4; ; 5IIH. (b) ESA–Zn^2+^, 30 mM Zn^2+^, pH 7.4; ; 5IJE. (c) ESA–Zn^2+^, 15 mM Zn^2+^, pH 6.5; ; 5IIX. Residues are shown in sticks representation, zinc ion in grey, oxygen in red, nitrogen in dark blue, carbon in green. Coordination bonds are marked with black dashed lines. Grey grid represents 2mf_o_ – df_c_ map (*σ* – 1.0), orange – anomalous map (*σ* – 3.0).

All six structures contained a Zn^2+^ ion bound in the location corresponding to site A (Table 3, site I and I′). In three structures, namely those with the lowest Zn^2+^ concentrations (; 5IJF, ; 5IIH, obtained by co-crystallization, and ; 5IIU, obtained after additional soaking, Table 1), the Zn^2+^ ion is bound in site A by His67, Asp249, and His247 (Fig. 2 and 3A), however in three other structures (; 5IIX, ; 5IJE, obtained by additional soaking, and ; 5IJ5, obtained by co-crystallization at low pH and the highest Zn^2+^ concentration), His247 is not involved in Zn^2+^ coordination due to a conformational change of its sidechain (Fig. 3B and C).

In the HSA structure, crystallized with 0.7 mM HSA and 0.5 mM Zn^2+^, site I is the only site that could be confidently modeled. The same is true for the ESA structure ; 5IIH crystallized with 0.2 mM ESA at pH 7.4 in presence of 2.5 mM Zn^2+^. It may be concluded that under near-stoichiometric and even slightly higher ratios of Zn^2+^ *vs.* serum albumin, only the strongest site(s) will become occupied, and that site I corresponds to the primary zinc binding site on HSA and ESA, *i.e.* site A. The site A found in the X-ray structures reported here adopts a tetrahedral geometry, involving three protein atoms: NE2 from His67, ND1 from His247, and OD2 from Asp249 plus a water molecule.

The all-atoms superposition of residues within 8 Å from Zn^2+^ in site A (16 residues, 110 atoms) in the HSA–Zn^2+^ complex (; 5IJF), and the corresponding residues in a ligand-free HSA structure (; 1AO6) shows only slight differences for the positions of the residues, as reflected in a low all-atom RMSD value of 0.4 Å (Fig. 2A). Similar calculation for native ESA (; 3V08) and ESA–Zn^2+^ complex (; 5IIH) shows a higher RMSD value of 1.2 Å (17 residues, 140 atoms). The higher RMSD value for ESA structures is caused by slightly different conformations of His67 and His247 sidechains participating in Zn^2+^ coordination and a nearby sidechain of Glu252, but the positions of C-alpha atoms remain the same. This finding indicates that site A in both HSA and ESA is mostly pre-organized in the fatty acid free form, however the adjustment of sidechain conformations may be necessary in order to coordinate Zn^2+^. The all-atom superimposition of residues within 8 Å from Zn^2+^ in site A in HSA–Zn^2+^ (; 5IJF) and ESA–Zn^2+^ (; 5IIH) also shows no significant structural differences (Fig. 2B) which suggests conservation of this site between the two organisms and probably other species, especially where residues coordinating Zn^2+^ are conserved (Fig. S2†).

The structures of serum albumin in complex with Zn^2+^ obtained at different ZnCl_2_ concentrations and pH illustrate the interesting dynamic behavior of the His247 sidechain (Fig. 3 and Table 3). In the structures of serum albumins with lower Zn^2+^ concentration (0.5–10.0 mM) and neutral or basic pH (PDB IDs: ; 5IJF, ; 5IIH, ; 5IIU), the His247 sidechain is involved in Zn^2+^ coordination in site A (Fig. 3A). In the structures of ESA with very high Zn^2+^ (30–50 mM) and/or proton concentration (PDB IDs: ; 5IJE, ; 5IJ5), the His247 sidechain adopts a different rotamer and is flipped away from the Zn^2+^ ion in site A and coordinates an additional Zn^2+^ ion (site XIV, Fig. 3B). In the structure of ESA obtained with 15 mM Zn^2+^ at pH 6.5, an intermediate state can be observed (; 5IIX): the sidechain of His247 is also flipped away from the Zn^2+^ in site A, however it is coordinating a water molecule instead of a Zn^2+^ ion (Fig. 3C). It is thus likely that the alternative His247 conformation in ESA–Zn^2+^ complexes is favored at lower pH and/or high Zn^2+^ concentration.

### Secondary and tertiary zinc binding sites

**Fig. 4:**
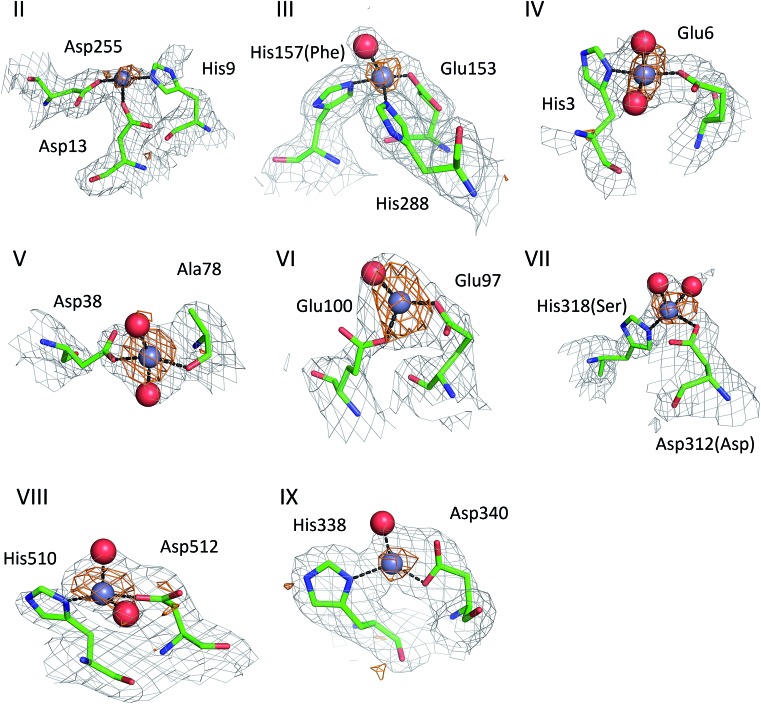
Secondary Zn^2+^ binding sites in the structures of ESA. Sites are numbered as in Table 3; only those coordinated by at least two protein residues are shown. Residues are shown in sticks representation, zinc ion in grey, oxygen in red, nitrogen in dark blue, carbon in green. Coordination bonds are marked with black dashed lines. Grey grid represents 2mf_o_ – df_c_ map (*σ* – 1.0), orange – anomalous map (*σ* – 3.0). Residue numbers refer to HSA; in case of non-conserved residues, the HSA amino acid is given in parentheses.

The structures of ESA–Zn^2+^ complexes that were soaked at final Zn^2+^ concentrations of 10 mM or higher revealed that besides the main “primary” Zn^2+^-binding site I (site A), many additional sites can be populated with Zn^2+^ (Table 3). All sites with complete coordination spheres adopted tetrahedral geometries (Fig. 4). Sites where Zn^2+^ is coordinated by three protein residues and likely bind Zn^2+^ strongly enough to contribute to its handling/carriage *in vivo* will be referred to as secondary sites. Sites coordinated by one or two sidechains and expected to bind Zn^2+^ more weakly are referred to as tertiary sites.

There are two secondary sites (II and III, Table 3) in ESA structures where Zn^2+^ is coordinated by three residues, like in site I. Site II is located at the highly disordered N-terminal part of serum albumin and was found only in one ESA structure (; 5IIU). In site II, Zn^2+^ is bound by one histidine residue and two carboxylates (His9, Asp13, and Asp255). Interestingly, in structures with higher zinc concentration, the anomalous signal for Zn^2+^ in this position was not present, and therefore Zn^2+^ was not placed in these structures. In the structure of HSA in complex with Zn^2+^, there is a weak anomalous signal present next to His9 and Asp13. However, the close proximity of a symmetric copy of the molecule in the HSA crystal (distance between His9 backbone and its asymmetric copy equals 4.0 Å) and location of the peak on the unit cell edge made it difficult to confidently model this Zn^2+^-binding site. Site III is located between domains IB and IIA and is present in all structures with 10 mM or higher Zn^2+^ concentration. Similarly to site A, site III is formed by two histidine residues (His157 and His288) and one carboxylate (Glu153). These residues are only partially conserved in mammalian albumins; His157 is replaced by Phe in HSA, suggesting weaker binding of Zn^2+^ in HSA in this location.

Of the six tertiary Zn^2+^ sites consisting of two residues (IV–IX), four are formed by a single histidine and a single carboxylate (Glu or Asp), one is formed by an aspartate residue and a main-chain oxygen (site V), and one is located on a crystallographic contact (site VI). Four of the tertiary sites consisting of two residues are conserved in HSA, but site VII is unlikely to be involved in Zn^2+^ binding in HSA due to substitution of His318 and Asp312 by Ser and Asn, which have no strong Zn^2+^-binding ability. In the crystals prepared with 10 mM or higher Zn^2+^ concentration, zinc was also located in several sites that have only one amino acid ligand (Table 3); these sites are not deemed to be of significance under physiological conditions. Interestingly, two of these sites (sites XII and XIII) have recently been implicated in Ru^3+^ binding.^29^ In none of the crystal structures was the N-terminal amine ordered, and no involvement in Zn^2+^ coordination was apparent; instead, His3 formed part of the two-residue site IV.

### Measurement of zinc binding to albumins by isothermal titration calorimetry (ITC)

**Fig. 5:**
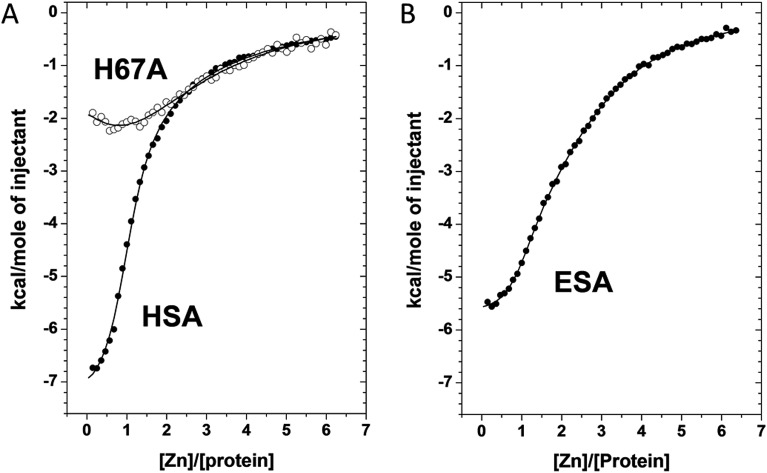
Measurement of Zn^2+^ binding affinity to HSA, H67A HSA mutant, and ESA by ITC. (a) ITC data for Zn^2+^ binding to HSA and the H67A mutant form. (b) ITC data for Zn^2+^ binding to ESA. In all cases the respective protein (50 μm) was titrated with 5 μl injections of a 1.5 mM ZnCl_2_ solution for 55 injections.

We used ITC to examine the Zn^2+^-binding properties of HSA, a mutant form of HSA (H67A), and ESA at near-physiological pH and ionic strength (50 mM Tris, 140 mM NaCl, pH 7.4). The resultant isotherms are shown in Fig. 5. Zn^2+^-binding to each protein under these conditions was exothermic. It should be noted that Tris possesses weak Zn^2+^-binding ability (log *K* = 2.27)^30,31^ which prevents formation and precipitation of Zn(OH)_2_ and thus ensures that Zn^2+^ remains in solution during the titrations. The competition from 50 mM Tris (which represents a 1000-fold excess over protein) also effectively prevents the detection of weak, non-physiological Zn^2+^ sites on the proteins. Thus only Zn^2+^-binding at reasonably strong sites (*K*_D_ < 10^–3^ M) can be observed under these conditions. Various models were explored to fit the resultant data corresponding to the binding of Zn^2+^ to HSA; a “two sequential sites” model fitted the data for HSA and H67A mutant well, with no substantial improvement of goodness-of-fit upon inclusion of a third binding site (Fig. 5A). This strongly indicates that under the experimental conditions, there are indeed only two sites with significant affinity in wild-type HSA, displaying a log *K*1_ITC_ value of 5.36 ± 0.05 and a log *K*2_ITC_ value of 3.8 ± 0.1, where *K*1 likely corresponds to site A, and *K*2 to one secondary site. Taking into account the competition with 50 mM Tris, these values can be converted into conditional dissociation constants, valid at the pH and ionic strength applied, giving *K*_D_1 = 1.7 ± 0.3 μM, and *K*_D_2 = 65 ± 21 μM. The values are in broad agreement with previous studies.^13,14^ Data fitting for the H67A mutant resulted in values for log *K*1_ITC_ = 4.64 ± 0.10 and log *K*2_ITC_ = 3.9 ± 0.1, giving *K*_D_1 = 9.1 ± 2.9 μM and *K*_D_2 = 51 ± 16 μM. The significantly decreased affinity to the first site indicates that the H67A mutation weakened binding to HSA by a factor of about 5. A similar conclusion had been reached previously by equilibrium dialysis.^9^ In contrast, the Zn^2+^-binding data for ESA were best fitted using a “three sequential sites” model (Fig. 5B), yielding log *K*1_ITC_ = 5.75 ± 0.10 (likely site A; *K*_D_1 = 0.7 ± 0.2 μM), log *K*2_ITC_ = 4.5 ± 0.1 (*K*_D_2 = 13 ± 4 μM) and log *K*3_ITC_ = 4.0 ± 0.1 (*K*_D_3 = 41 ± 13 μM). This suggests that ESA harbors an additional Zn^2+^-binding site compared to HSA, with its affinity lying between that of site A and the second HSA site.

## Discussion

The importance of albumin in Zn^2+^ handling and transport has been widely studied;^18^ however, the locations and structures of individual Zn^2+^-binding sites on the albumin molecule have so far only been inferred indirectly. Here we present the first crystal structures of two serum albumins in complex with Zn^2+^. Our crystallographic studies provide new insights into the geometry of the main high-affinity Zn^2+^-binding site on albumin (site A) and identify several secondary and tertiary Zn^2+^-binding sites. Additionally, examination of structures corresponding to albumins at different pH values and in the presence of various Zn^2+^ concentrations provides insight into the structural dynamics of a crucial inter-domain histidine residue, His247, located at the domain I/II interface.

### Site A: location, structure, and dynamics

Site A has long been thought to be the strongest Zn^2+^-binding site on albumin,^15^ and its location has previously been pinpointed through site-directed mutagenesis.^20^ Our studies further support this notion by the observation that the corresponding site is occupied by Zn^2+^ in all presented structures, even when crystallized in the presence of sub-stoichiometric zinc concentrations (*e.g.* in HSA). The crystallographic data reveal that the geometry and residues involved in Zn^2+^ coordination at site A are conserved between HSA and ESA. Inspection of albumin protein sequences from 14 different mammalian species suggests high conservation of this site across mammals (Fig. S2†).

The structures obtained at neutral or basic pH and lower zinc concentrations (5IJF and ; 5IIH obtained by co-crystallization, and ; 5IIU obtained by additional soaking, Table 1) likely best reflect the situation in physiological conditions. Comparison of these structures with their metal-free equivalents testifies to the high degree of pre-organization of site A (Fig. 2). The tetrahedral Zn^2+^ coordination sphere of site A observed in these crystal structures (inner-sphere ligands: sidechains of His67, His247, Asp249, and a water molecule) shares three protein residues with previously proposed models.^9,20^ The previous models and the structures presented here differ in the presence/absence of coordinative bonds to the sidechain OD1 of Asn99 and the backbone carbonyl oxygen of His247 that were included in the model based on interpretation of EXAFS data for wild-type and mutant HSA and optimization by force-field-based energy minimization. These differences are brought about mainly by the different position of the Zn^2+^ ion; a direct comparison between the serum albumin–Zn^2+^ crystal structures and the model reveals that the predicted location of the Zn^2+^ ion differs by 2.05–2.11 Å from that found by structure determination (Fig. S3†). In consequence, the amide OD1 atom of Asn99 is at a distance of 3.9–4.4 Å and the backbone O of His247 at 3.6–4.8 Å from the Zn^2+^ in all studied crystal structures. Both distances are too long for coordinative bonds, although charge–dipole interactions cannot be ruled out. We considered the possibility for outer-sphere coordination, *i.e. via* a Zn^2+^-bound water molecule, but the electron density maps did not support this possibility.

The ability of carbonyl oxygens from an amide or ester group to coordinate Zn^2+^ is well-documented,^32^ especially during ester and amide hydrolysis. Such bonds are both relatively weak and labile, and indeed, replacement of Asn99 by His or Asp has been shown to strengthen zinc binding to albumin about 10-fold.^9^ Whilst this does not allow inferring Asn99 coordination in the wild-type, we speculate that zinc binding to site A may be more dynamic than reflected by either structure or model, a suggestion that is in keeping with the outstandingly high flexibility of albumin in general. The tetrahedral site observed here resembles a classical catalytic site found in many hydrolytic enzymes.^33^ Despite earlier suggestions that metal transport sites should substantially differ from catalytic sites^34^ due to their fundamentally different mechanistic roles, many recent structural studies on zinc transport proteins have, surprisingly, revealed transport sites that similarly resembled catalytic sites.^35^

The structures obtained at high zinc and/or proton concentrations (5IIX and ; 5IJE obtained by additional soaking, and ; 5IJ5 obtained by co-crystallization, Table 1) have revealed a further dynamic aspect of site A that is located at the domain I/II interface: His247 can exist in at least two different conformations (Fig. 3). In the structures with low zinc and/or proton concentration, His247 participates in zinc coordination in site A. With increasing zinc and/or proton concentration, His247 flips away from site A and either interacts with a water molecule, or coordinates an additional zinc ion. Protonation of His247, or over-saturation with Zn^2+^, seems to favor a conformation in which the sidechain is flipped away from Zn^2+^ in site A. We do not anticipate that the “flipped-away” conformation plays any significant role in physiological zinc-bound states of albumin, but speculate that it has significance in the context of the so-called neutral-to-basic (N-to-B) transition. In the majority of published albumin structures, the sidechain of His247 forms a H-bond to that of Asp249, which in turn has a H-bond with His67. The interaction between these three residues (which incidentally also form the zinc binding site A) thus stabilizes the domain I/II interface, which otherwise has a scarcity of other stabilizing electrostatic or non-covalent interactions. The dynamic behavior of His247, pointed out on the basis of ESA–Zn^2+^ structures, is also observed in previously published native structures of serum albumin, but without direct correlation with pH. For example in ESA, His247 is H-bonded to Asp249 in one reported X-ray crystal structure (; 4F5T, pH 4.5)^24^ (the pH values for these structures were taken as reported in PDB entries and do not necessarily reflect final pH in the crystallization drops),^36^ but is flipped away in three other structures (PDB IDs: ; 3V08, pH 7.4; ; 4F5S, pH 6.5; ; 5HOZ, pH 9.0).^24,25^ Likewise for HSA, the His247-Asp249 H-bond is present in the structure ; 1AO6 at pH 7.5,^27^ but the sidechain of His247 points away from Asp249 in ; 4K2C at pH 7.0.^37^ It has been known for a long time that albumin undergoes various pH-dependent conformational transitions, and that the N-to-B transition is mediated by the protonation state of domain-interface histidine residues, as the only residues with p*K*_a_ values in the neutral range.^38,39^ This “conformational transition” should not be envisaged as a large change in the mutual orientation of entire domains, but rather as a subtle change in conformational dynamics at the domain I/II interface. As one of the very few H-bonding residues at this interface, His247 is likely a key player in these dynamics. The binding of several ligands to albumin, including drugs and fatty acids, have been shown to depend on the state of the N–B equilibrium, due to differences in affinity of the N and B states.^40^ This is likely to be of importance physiologically, not least in pathological conditions associated with alkalosis, including hypoalbuminemia.^41^

### Secondary zinc binding sites: determining the location of site B

We have previously demonstrated that site A undergoes significant disruption upon binding of fatty acids to albumins (HSA and BSA) such that the Zn^2+^ binding affinity of this site is significantly affected in the presence of physiological (and pathophysiological) concentrations of fatty acids.^6,14^ In addition, we have shown that there is an overlap in residues involved in Ca^2+^ binding.^25^ These observations raise the question how Zn^2+^ binding changes when site A is compromised and underlines the biological importance of additional Zn^2+^-binding site(s) on serum albumin. The structures corresponding to crystals grown in the presence of higher Zn^2+^ concentrations give insight into the possible locations of these Zn^2+^ sites. Zn^2+^ was found associated with ESA in 17 different locations, in addition to site A, which is in agreement with results of gel filtration chromatography.^23^ In 15 of the 17 sites, Zn^2+^ is coordinated by only one or two protein residues (called herein tertiary sites) and due to their expected lower binding affinity, these are not likely to have an impact on Zn^2+^ transport *in vivo*.

In addition to the main site A and 15 tertiary sites, two secondary Zn^2+^-binding sites were located in ESA–Zn^2+^ structures (Table 3). Our own ITC measurements, as well as various literature reports^9,16,21,23^ suggest that in solution, there are only either one or two zinc binding sites with significant affinity besides site A in HSA or BSA. We suggest that these additional sites correspond to secondary sites in ESA–Zn^2+^ crystal structures. The secondary sites provide additional Zn^2+^-binding capacity on serum albumin and likely contribute to Zn^2+^ handling/transport in the circulation, particularly when site A is disrupted. Historically, this capacity has been ascribed to the so-called site B which is suggested to be the second strongest Zn^2+^-binding site on HSA and BSA.^4^ There has been much uncertainty about the location of site B. Out of two secondary sites (sites II and III), only one of them, site II (composed of His9/Asp13/Asp255) is conserved in HSA and BSA. The coordination sphere of Zn^2+^ then consists of one nitrogen atom from His and two oxygen atoms from Asp, in broad agreement with ^113^Cd^2+^ NMR data that predicted one or no nitrogen ligand with otherwise all-oxygen coordination for site B.^17^ Considering the observation of weak anomalous electron density near His9/Asp13 in the HSA structure at low zinc concentration, together with the presence of this site in the ; 5IIU ESA structure, this site appears as a particularly attractive candidate for site B. Intriguingly, this site would also be an inter-domain site, and indeed, previous ITC measurements and ^111^Cd NMR spectroscopic studies^13^ on BSA indicated that the secondary zinc site, and Cd site B, were also affected by myristate binding. Two of the corresponding site II residues in BSA (Asp13 and Asp254) have also previously been implicated in Ca^2+^ binding.^24^ This suggests that cross-talk may exist between Zn^2+^ and Ca^2+^ binding at this site, similarly to site A.

Site III in ESA is also formed by three protein ligands (composed of His157/His288/Glu153), similar to site A and site II, but this site is not conserved in HSA. Our ITC measurements indicated that ESA contains one binding site more than HSA; we suggest that this extra site corresponds to site III, and, based on the magnitude of the *K*_D_ values, that it is associated with *K*_D_2. Site III has similar coordination and geometry (two His, one Asp residue, and one water molecule in tetrahedral geometry) as site I, but much weaker affinity for zinc (0.7 μM in site I *vs.* 13 μM in site III). The observed differences may be attributed to differences in the local environment. In site I, there is a negatively charged Glu252 residue within 6 Å from the Zn^2+^-binding site that will electrostatically stabilize the positively charged metal ion in site I. In addition, the previously mentioned carbonyl oxygens from the backbone of His247 and the amide sidechain of Asn99 may also contribute to stability through electrostatic interactions. Conversely, site III is in the vicinity of two positively charged residues, Arg188 (Glu in HSA) and Arg257. Arg188 is within 6 Å from the Zn^2+^-binding site, which may cause electrostatic repulsion. Arg257 is also within 3.5 Å from Glu153 that coordinates Zn^2+^ in site III. The interaction between Arg257 and Glu153 is likely to reduce the affinity of Glu153 for Zn^2+^. Interestingly, one of the residues forming site III (His288) has also been implicated in binding of Pt^2+^ to HSA.^42^

### ATCUN motif and free Cys34: no structural evidence for zinc binding

Several metal-binding sites on albumins have been described in the literature; in particular, the N-terminal ATCUN (amino-terminal copper- and nickel-binding) motif^43^ and the free thiol at Cys34,^4^ but neither was seen to play a role in Zn^2+^ binding in our crystal structures. The N-terminus in albumins is very flexible which impedes the interpretation of crystallographic data (the first two or three residues cannot be built). The first three residues of most albumin sequences, *e.g.* Asp1-Ala2-His3 in HSA, form the aforementioned ATCUN motif, observed in several different proteins. The ATCUN motif is particularly suitable for coordination of metal ions with a preference for tetragonal or square planar geometry like Cu^2+^ and Ni^2+^, with concomitant deprotonation of two backbone amide nitrogens.^44^ In principle, and as shown by studies involving tripeptides, other metal ions, including Zn^2+^ and Co^2+^, can also bind to an ATCUN motif, but much more weakly, and only at much higher than physiological pH.^45^ Although Zn^2+^ was found bound to His3 (site IV), the respective site also involved Glu6 rather than the N-terminus or backbone amides, further emphasizing that caution must be applied when interpreting metal-binding data from short peptides.^46^

Another site that might have been expected to be observed associated with Zn^2+^ is the free thiol at Cys34, as cysteine is a common Zn^2+^-binding ligand in protein structures. However, in none of our structures did we observe zinc-related electron density near Cys34. The Ellman's reagent assay showed 0.27 molar equivalents of free cysteine (which refers to Cys34 since it is the only cysteine not involved in disulphide bonds). Therefore, it is possible that binding was prevented by cysteine oxidation, although in none of the structures any electron density for the respective oxygen atoms was observed. We note that it has been known for some time that Cys34 is not a major (primary or secondary) binding site for Zn^2+^ on HSA.^17^ More recent work on the HSA C34A mutant also demonstrated clearly that Cys34 is not required for either site A or B.^9^ The apparent inability of Cys34 to form a significant Zn^2+^-binding site is most likely due to a lack of other potential metal-coordinating residues located nearby. In accordance with this, Cys34 tends to be associated more with binding of complexes of very soft metal ions rather than 3d row metal ions; these include complexes containing Au^+^ and Pt^2+^.^47,48^ Even so, Cys34 was also not observed as a Pt^2+^ binding site in a recent crystallographic study.^42^ Given our observations, it is conceivable that the Zn^2+^–single thiolate interaction may be too weak to be observed.

### Potential influence of soaking on zinc binding

Additional crystal soaking after initial co-crystallization (5IIU, ; 5IIX and ; 5IJE) allowed for higher Zn^2+^ concentration in the drop while maintaining neutral pH, which, in turn, revealed several additional Zn^2+^-binding sites that could not be detected at lower Zn^2+^ concentrations. Soaking is a very popular technique used to introduce ligands into proteins, and it is assumed to produce essentially the same results as compared to co-crystallization when the final concentration of the ligand is the same. Nevertheless, one should keep in mind that it may possibly result in a different mode of ligand binding (as compared to co-crystallization) for various reasons. (1) The ligand may not have access to the binding site in the crystalline form. However, this issue is highly unlikely with small ligands like Zn^2+^ that can easily penetrate the crystal. (2) Some Zn^2+^-binding sites located on the surface of the protein may be blocked during soaking due to crystal contact formation. In albumin, however, only 10% of the protein surface area is involved in crystal contacts as calculated using the PISA server.^49^ This is much lower than the average surface area buried by crystal contacts in protein crystal structures, which ranges from 15–49%.^50^ Taken together, this suggests that the majority of the surface in albumin crystals is not involved in crystal contacts, and so the accessibility of most Zn^2+^-binding sites should not be affected by the crystal lattice formation. (3) The formation of the lattice could prevent necessary conformational changes and hinder Zn^2+^ binding in some sites. This may apply to sites located near the crystal contacts (which is a rare case for albumin as mentioned in point 2) or to sites which require massive conformational changes in order for Zn^2+^ to bind. All the other sites should possess the same conformational freedom (at least for amino-acid sidechains) in the crystal and in the solution, as was manifested by the conformational dynamics of His247 (*vide supra*). Taking all these considerations into account, we assume that observations made for soaked crystals – such as flipping of His247 and presence of additional Zn^2+^-binding sites – were a result of increased Zn^2+^ concentration, not the experimental procedure used to achieve the final Zn^2+^ concentration.

### Zinc homeostasis in blood plasma

The dissociation constants determined by ITC under near-physiological conditions for the major zinc binding site in the low micromolar range, together with average concentrations for albumin and exchangeable zinc in plasma, allow estimating the free Zn^2+^ concentration as lying in the tens of nanomolar, if no other binding partners are considered. Although much higher than intracellular free Zn^2+^ concentrations, this extracellular concentration is low enough to avoid cell toxicity. At the same time, the binding of Zn^2+^ to albumin is both thermodynamically relatively weak and kinetically labile, and this facilitates dissociative Zn^2+^ transfer to other binding partners including cellular zinc uptake transporters and other plasma proteins.^6^ Thus, the extracellular Zn^2+^ buffer albumin regulates the availability of plasma Zn^2+^ for other processes. Crucially, previous work has shown that the Zn^2+^-binding capacity of albumin is dependent on the presence of other metabolites, most importantly free fatty acids.^13,14^ Binding of these in fatty-acid binding site FA2^51^ disrupts zinc site A (Fig. S4†). Under these conditions, the secondary site(s) become significant; speciation modeling for human plasma has shown that due to the high concentration of albumin, its secondary sites still provide a relevant Zn^2+^ buffer. Even so, the free Zn^2+^ concentration was estimated to increase into the low micromolar range (≤1 μM) at the highest levels of fatty acid overload.^14^ Clearly, such an increase in non-albumin bound zinc is likely to increase Zn^2+^ association with other plasma proteins, and lead to increased cellular uptake, with multiple downstream effects.

## Conclusions

The adequate distribution of the essential nutrient Zn^2+^ throughout the body and its delivery to all cells is of fundamental importance. Here, for the first time we have illustrated details of Zn^2+^–albumin binding on the atomic level using X-ray crystallography. It is at these identified sites that the speciation and distribution of Zn^2+^ in the blood is controlled. In addition to metal ions, serum albumin also transports other physiologically important molecules such as fatty acids, hormones and drugs, with albumin emerging as a central hub in plasma that links the distribution of these major metabolites.^6,14,52^ At present there are over 120 albumin structures (predominantly of HSA) available in the RCSB Protein Databank. Collectively these structures contain information pertaining to conformational effects triggered by the binding of various small molecules to albumins. Establishing the precise location and properties of the main physiologically-relevant Zn^2+^ sites allows assessment of how binding of other molecules influences Zn^2+^ carriage by albumin. Such interplay is likely to regulate the activity of other Zn^2+^-binding plasma proteins, such as histidine-rich glycoprotein, influence cellular Zn^2+^ uptake, or in extreme cases could lead to cell damage due to Zn toxicity. Thus the presented data allow unprecedented insight into Zn^2+^ binding at several sites on the albumin molecule, providing a platform for exploring the dynamic interplay between Zn^2+^ and other physiologically important molecules transported by serum albumin.

## Experimental

### Mutation, expression, purification and crystallization

The coding sequence of human serum albumin (corresponding to residues 19-609 of the prepro-albumin sequence; NP_000468) was amplified and cloned into pKLAC2 plasmid using *Xho*/*NotI* restriction sites downstream and in frame with the α-mating factor secretion signal sequence. Oligonucleotide directed mutagenesis was used to prepare a construct encoding the mutated albumin (H67A) using the QuikChange Site-Directed Mutagenesis Kit (Agilent, Cheshire, UK). The resultant plasmids were linearized using *BstXI* enzyme and transformed into *Kluyveromyces lactis* GG799 competent cells (New England Biolabs, Hitchin, UK) in accordance with the manufacturer's instructions and grown under acetamide selection. Genomic DNA was extracted from resultant clones using the Wizard Yeast Genomic DNA Purification Kit (Promega, Southampton, UK) and correct insertion of the expression cassette into the *K. lactis* genome was verified by PCR, using primers flanking the insertion site. A colony expressing the required protein of interest was grown overnight in 50 ml of sterile YPGlu medium (1% yeast extract, 2% Bacto-Peptone, 2% glucose) at 30 °C with shaking at 200 rpm. This starter culture was used at ratio of 1/100 to inoculate 4 L of sterile YPGal (1% yeast extract, 2% Bacto-Peptone, 2% galactose). The culture was grown for 3 days at 30 °C with shaking at 200 rpm at pH 4.5–5.5. Finally, the supernatant was removed following centrifugation and purification proceeded by applying a concentrated sample of supernatant on HiTrap Blue HP (GE Healthcare, Little Chalfont, UK). This procedure was followed by anion exchange (HiTrap DEAE FF; GE Healthcare) and size exclusion (HiLoad Superdex-75 column; GE Healthcare) techniques. All preparations were performed on an ÄKTA Purifier (GE Healthcare). Final purity of protein by SDS-PAGE was >95%. Protein was dialyzed in 50 mM Tris, 140 mM NaCl, pH 7.4 prior to ITC Zn^2+^-binding experiments.

For crystallization, ESA was purchased from Equitech-Bio (Kerrville, TX, LOT# ESA62-985, Catalog# ESA62) and HSA from Sigma-Aldrich (St. Louis, MO, LOT# SLBD7204, Catalog# A3782), both as defatted lyophilized powder. ESA was dissolved in 10 mM Tris pH 7.5 and 150 mM NaCl buffer and HSA was dissolved in 20 mM K_2_HPO_4_ pH 7.5 buffer. Proteins were purified using a Superdex 200 gel filtration column attached to an ÄKTA FPLC system (GE Healthcare) at 4 °C. Following gel filtration, fractions containing monomeric proteins with the molecular weight of 55–60 kDa were selected and concentrated to 30 mg ml^–1^ for ESA and 90 mg ml^–1^ for HSA.

ZnCl_2_ (Sigma Aldrich, chemical grade ≥98%) was used to prepare crystallization conditions and stock solutions for crystals soaking. Since ZnCl_2_ is known to decrease pH of water solutions due to formation of Zn(OH)_2_, a special care was taken in order to control the pH of the final solutions. To assure the final pH in crystallization drop to be as intended (*e.g.* 4.5, 6.5, 6.9, 7.0, 7.4, 9.0), the pH in every zinc-containing crystallization condition was adjusted with NaOH prior to crystallization experiment. The solution of ZnCl_2_ used for crystals soaking was prepared by dissolving ZnCl_2_ in 100 mM Tris buffer to final concentration of 100 mM. The pH was adjusted to a chosen value.

In order to obtain diffracting crystals of HSA–Zn^2+^ complexes, the sitting-drop vapour diffusion method was used on triple drop, 96-well crystallization plates from Hampton Research (Aliso Viejo, CA). Plates were set up using a Mosquito crystallization robot (TTP Labtech, Cambridge, MA). Both the standard setup and alternative reservoir methods^53^ were used with more than 10 commercially available screens. All of the crystallization trials were attempted with varied Zn^2+^ (1–20 mM) and protein concentrations (70–240 mg ml^–1^), as well as with protein : reservoir ratios of 1 : 1, 1 : 2 and 2 : 1. Crystals suitable for X-ray experiments grew only in condition no. 20 from the Wizard IV screen (25% (w/v) PEG 1500, 100 mM MMT buffer pH 9.0) in the presence of 0.5 mM ZnCl_2_ (Table 1). In addition, crystallization conditions of HSA structures deposited in the PDB were also tried with grid-screening optimizations by hanging-drop vapour diffusion method on 15-well plates (Qiagen, Valencia, CA). Crystals of HSA–Zn^2+^ complexes and native HSA (for soaking experiment) were grown using PDB conditions, but they did not diffract better than 4 Å.

For ESA–Zn^2+^ complexes, more than 10 commercial screens were tested using the sitting-drop vapour diffusion method with the standard setup on 96-well plates. Screening gave several conditions with diffracting crystals. We selected two of them, one at acidic pH (2.0 M ammonium sulfate, 100 mM sodium acetate pH 4.5) and one at close to neutral pH (200 mM lithium sulfate, 100 mM Tris:HCl, pH 7.0, 2.0 M ammonium sulfate) that were used for further optimization of ESA–Zn^2+^ complexes. Crystals were obtained by the hanging-drop vapour diffusion method on 15-well plates (Qiagen, Valencia, CA). Plates were set up manually with a 1 : 1 protein : reservoir ratio. All ESA–Zn^2+^ complexes were crystallized in the presence of 2.5 or 50 mM ZnCl_2_. Additionally, selected crystals of ESA–Zn^2+^ complexes were soaked at higher ZnCl_2_ concentrations by adding 0.5–2.5 μl of 100 mM ZnCl_2_ in 100 mM Tris (Table 1). Crystallization and cryo-protectant conditions are summarized in Table 1.

### Data collection and anomalous scattering

Data were collected from single crystals at 100 K at the LS-CAT 21-ID-F for HSA–Zn^2+^ and for ESA–Zn^2+^ at LS-CAT 21-ID-G and SBC-CAT 19-BM beamlines at the Advanced Photon Source, Argonne National Laboratory (Argonne, IL). Diffraction images for HSA–Zn^2+^ and ESA–Zn^2+^ were collected at the selenium absorption K-edge (12 664 eV), where anomalous scattering on Zn^2+^ is strong. The selected crystal of ESA in 30 mM ZnCl_2_ (; 5IJE) was scanned for X-ray fluorescence emission in order to confirm identity of the metal. Additionally, the identity of the bound metal for this crystal was confirmed by comparing diffraction data collected on wavelengths corresponding to energies below (9618 eV) and above (9668 eV) the zinc absorption K-edge (9659 eV, Fig. S1†).

### Structure determination and refinement

Structures of HSA and ESA were solved by molecular replacement using native structures of both proteins (1AO6 for HSA and ; 3V08 for ; 5IIH and afterwards ; 5IIH for the other four ESA structures) previously deposited in the PDB as template models. The data were integrated and scaled by HKL-3000^54^ using scale anomalous mode, enabling calculation of an anomalous map during subsequent structure refinement. Molecular replacement was performed using MOLREP^55^ and other programs from the CCP4 package^56^ incorporated into HKL-3000. The refinement was performed with REFMAC^57^ in HKL-3000. COOT^58^ was used for manual model inspection and real-space refinement. TLS was used in the final stages of the refinement, and TLS groups were introduced using the TLSMD server.^59^ A stand-alone version of MOLPROBITY^60^ incorporated into HKL-3000 and the Worldwide PDB (wwPDB) validation server^61^ were used for structure quality assessment. Zn^2+^-binding sites were additionally validated using the CheckMyMetal server.^62^ Data collection and refinement statistics are summarized in Table 2. The atomic coordinates and structure factors have been deposited in the PDB with identifiers: ; 5IJF, ; 5IIH, ; 5IIU, ; 5IIX, ; 5IJE and ; 5IJ5. The diffraction images are available on Integrated Resource for Reproducibility in Macromolecular Crystallography website (; http://proteindiffraction.org/) with DOI: ; 10.18430/M35IIH, DOI: ; 10.18430/M35IIU, DOI: ; 10.18430/M35IIX, DOI: ; 10.18430/M35IJ5, DOI: ; 10.18430/M35IJE, DOI: ; 10.18430/M35IJF.

### Isothermal titration calorimetry (ITC)

Binding of Zn^2+^ to HSA, H67A mutant HSA, and ESA was assessed by ITC. In each case experiments were performed using a MicroCal VP-ITC instrument (GE Healthcare). Proteins were dialyzed in 50 mM Tris, 140 mM NaCl, pH 7.4 prior to analysis. Prior to titration ZnCl_2_ was added to the reaction buffer and the pH re-adjusted to 7.4. Samples were degassed for 15 minutes. All experiments were performed at 25 °C with 50 μM albumin samples and 1.5 mM ZnCl_2_. The parameters for each ITC experiment were set to one injection of 2 μl over 4 s followed by up to 55 injections of 5 μl over 10 s with an interval of 240 s between injections to allow complete equilibration. The stirring speed was set to 307 rpm. Heats of dilution were accounted for with blank titrations performed by titrating the ZnCl_2_ solution into reaction buffer and subtracting the averaged heat of dilution from the main experiments. Raw data were processed using MicroCal Origin software and data were fitted using the same software.

### Sequence and structure analysis

Homology sequence search was performed using BLAST^63^ against UniProtKB database with default options. Sequences were aligned using the MAFFT server^64^ with default parameters. The SSM algorithm^65^ implemented in COOT was used to superpose structures and calculate RMSD values.
