# Supplementary material for: Circulatory zinc transport is controlled by distinct interdomain sites on mammalian albumins
Source: Chem Sci. 2016 Aug 15;7(11):6635–48. doi: 10.1039/c6sc02267g (PMC5450522; doi:10.1039/c6sc02267g)
Supplement: Supplementary file 1 [file SC-007-C6SC02267G-s001.pdf]

Electronic Supplementary Material (ESI) for Chemical Science.
This journal is © The Royal Society of Chemistry 2016

Supplementary information

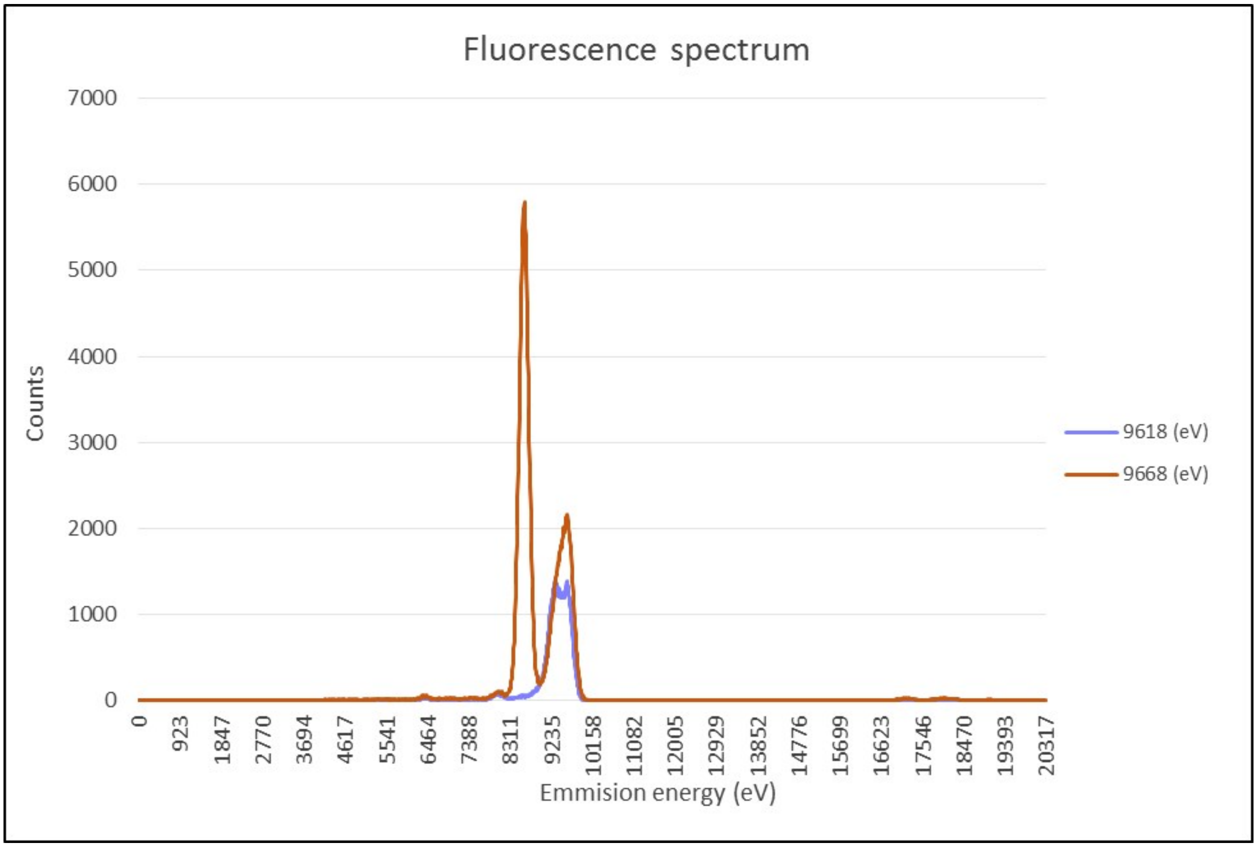

Figure S1. Excited fluorescence spectrum of zinc scattering at energies below (9618 eV – blue) and above (9668 eV –brown) zinc absorption K-edge.

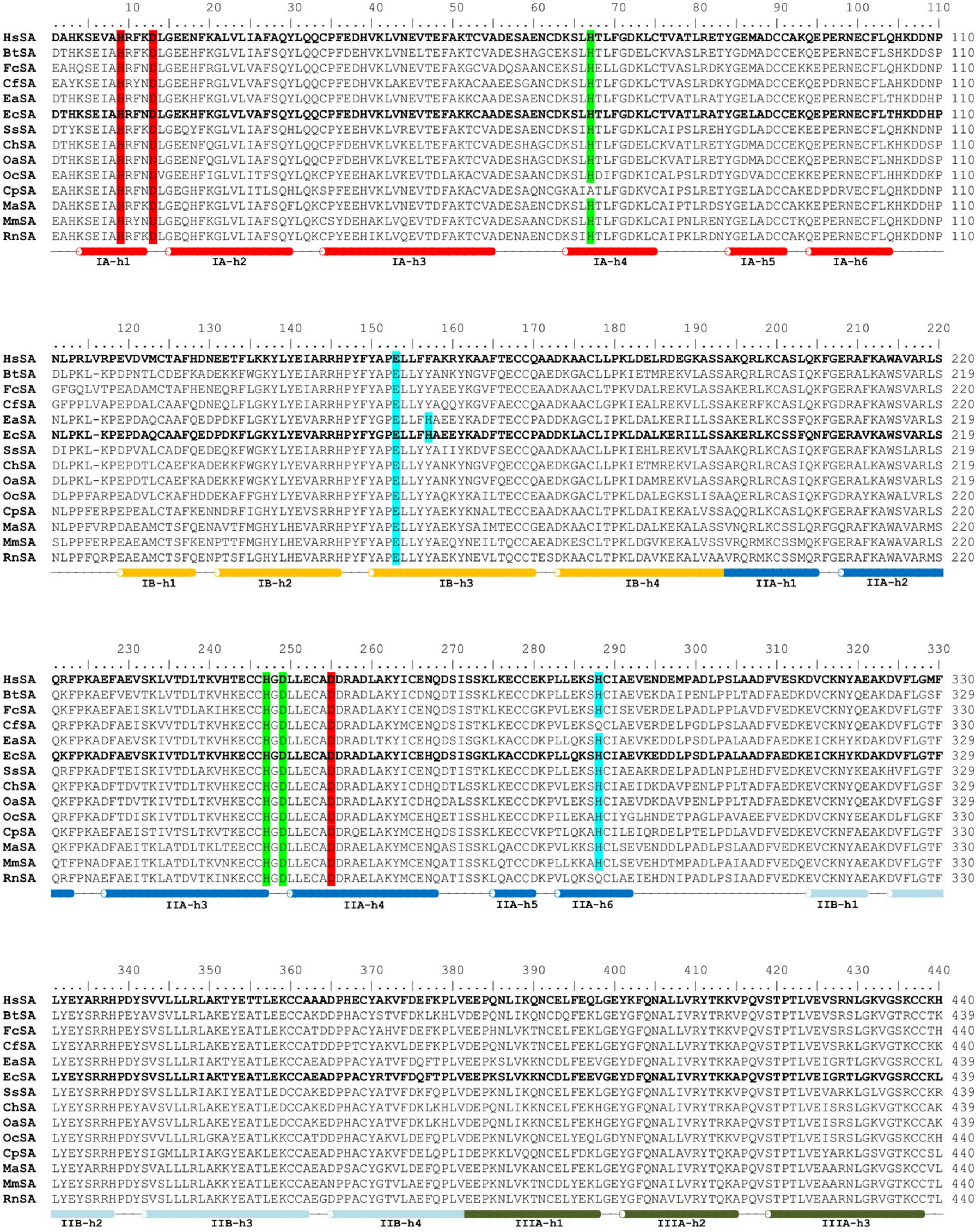
HsSA
BtSA
FcSA
CfSA
EaSA
EcSA
SsSA
ChSA
OaSA
OcSA
CpSA
MaSA
MmSA
RnSA
10 20 30 40 50 60 70 80 90 100 110
IA-h1 IA-h2 IA-h3 IA-h4 IA-h5 IA-h6
120 130 140 150 160 170 180 190 200 210 220
IB-h1 IB-h2 IB-h3 IB-h4 IIA-h1 IIA-h2
230 240 250 260 270 280 290 300 310 320 330
IIA-h3 IIA-h4 IIA-h5 IIA-h6 IIB-h1
340 350 360 370 380 390 400 410 420 430 440
IIB-h2 IIB-h3 IIB-h4 IIIA-h1 IIIA-h2 IIIA-h3

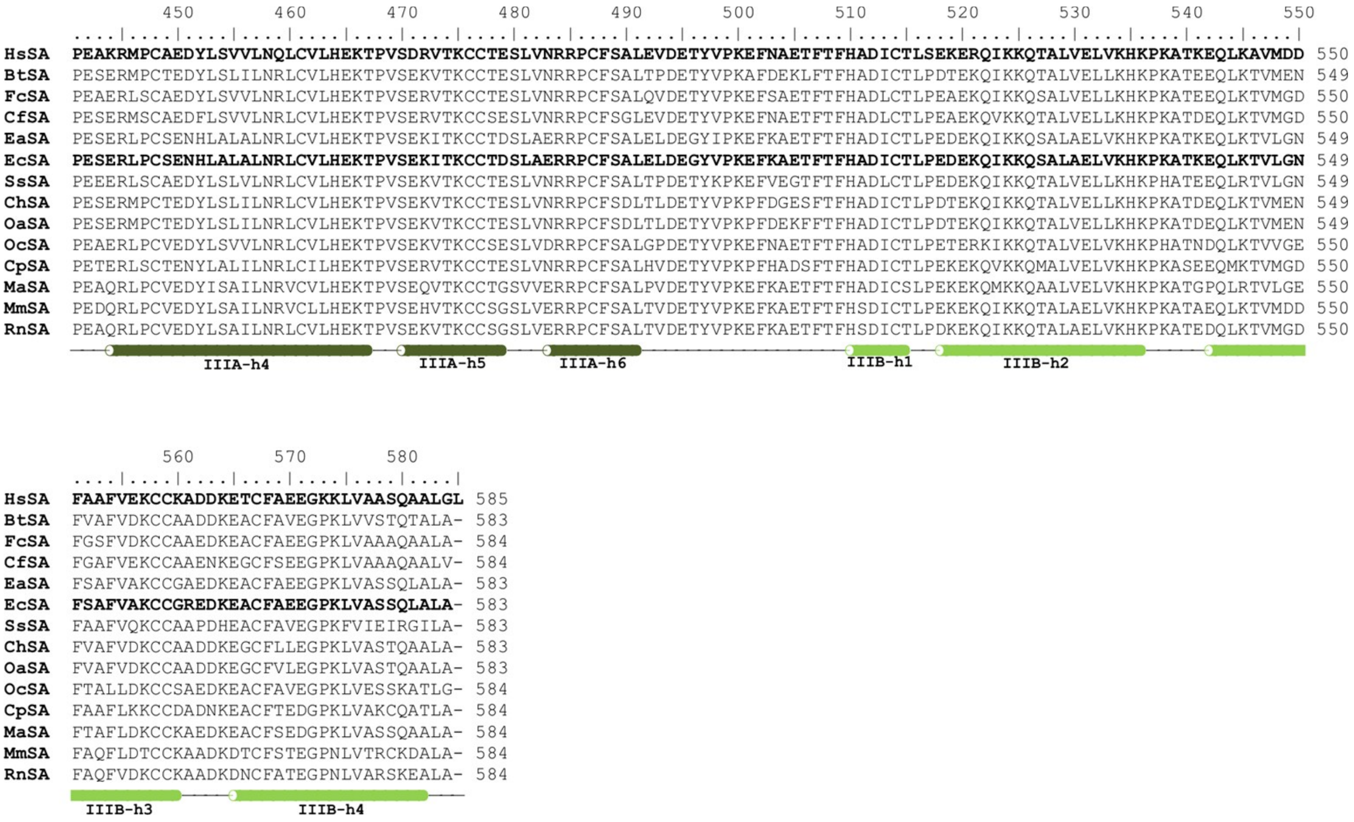

Figure S2. Conservation of amino acid residues involved in site A (I) (green highlights), site II (red highlights), and site III (turquoise highlights) in mammalian albumins. Sites are numbered as in Table 3. Alignments were carried out using MAFFT server.[1] Human and Equine albumins are shown in bold. Protein sequence UniProt accession numbers:

HsSA - Homo Sapiens – human - P02768; BtSA - Bos taurus – bovine - P02769; FcSA - Felis cattus – cat - P49064; CfSA - Canis familiaris – dog - P49822; EaSA - Equus asinus – donkey - Q5XLE4; EcSA - Equus caballus – horse - P35747; SsSA - Sus scrofa – pig - P08835; ChSA - Capra hircus – goat - P85295; OaSA - Ovis aries – sheep - P14639; OcSA - Oryctolagus cuniculus – rabbit - P49065; CpSA - Cavia porcellus – guinea pig - H0V003; MaSA - Mesocricetus auratus – golden hamster - A6YF56; MmSA - Mus musculus – mouse - P07724; RnSA - Rattus norvegicus – rat - P02770

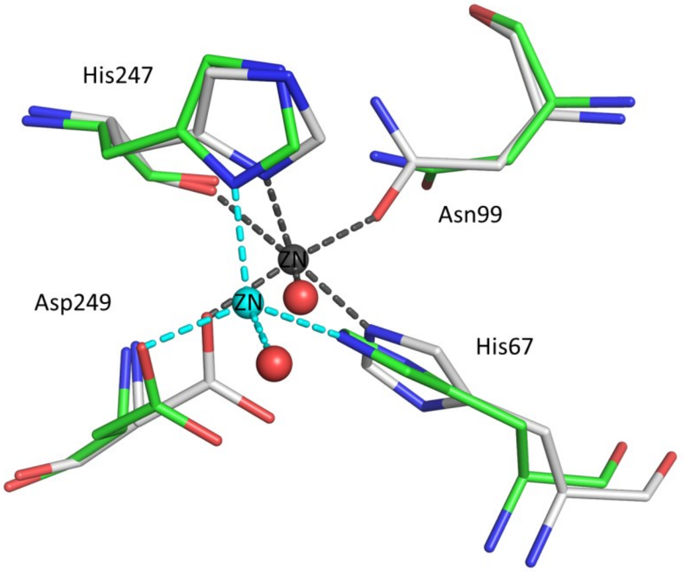

Figure S3. Superposition of EXAFS model (carbon atoms white) of HSA[2] in complex with zinc and crystal structure of ESA (this work) in complex with zinc (carbon atoms green, PDB ID: 5IIH). Nitrogen is marked in blue, oxygen in red. Zinc is presented in dark gray in the EXAFS structure and in cyan in crystal structure. Bonds between metal and coordinating atoms are represented by dashed lines.

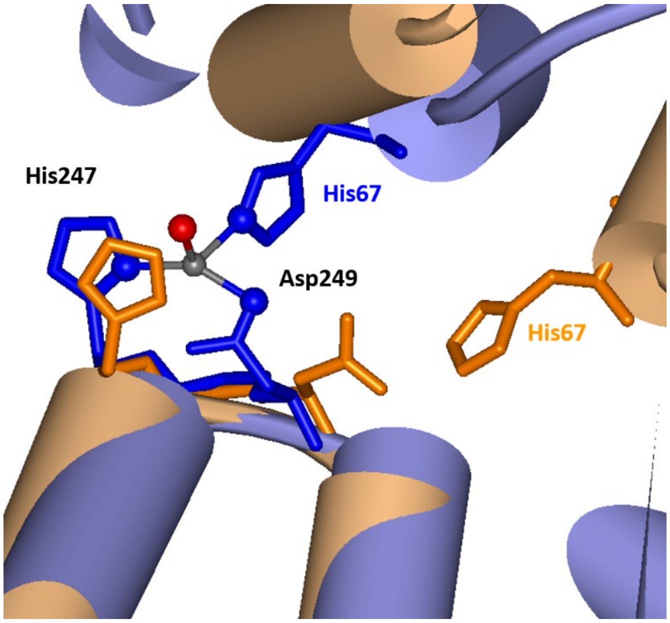

Figure S4. Fatty-acid binding induced disruption of the major zinc binding site on human serum albumin. Residues and backbone schematics in blue refer to pdb 5IJF (this work), residues and backbone in orange refer to pdb 1BJ5.[3] The zinc ion is shown in grey, and the coordinated water oxygen in red. A domain-domain movement induced by binding of myristate in fatty acid binding site FA2 increases the distance between the zinc-coordinating nitrogen atoms from 3.5 Å to 8.6 Å. The overlay was generated in Swiss pdb viewer v. 4.1 by matching the backbone atoms for residues 245-251. The Figure was generated in WebLabViewerpro 4.0.

References

1 K. Katoh and D. M. Standley, *Mol. Biol. Evol.*, 2013, **30**, 772–80.

2 C. A. Blindauer, I. Harvey, K. E. Bunyan, A. J. Stewart, D. Sleep, D. J. Harrison, S. Berezenko and P. J. Sadler, *J. Biol. Chem.*, 2009, **284**, 23116–24.

3 S. Curry, H. Mandelkow, P. Brick and N. Franks, *Nat. Struct. Biol.*, 1998, **5**, 827–835.
